# Impact of Zinc Oxide Addition on Oil Palm Empty Fruit Bunches Foamed Polymer Composites for Automotive Interior Parts

**DOI:** 10.3390/polym15020422

**Published:** 2023-01-13

**Authors:** Taufan Arif Adlie, Nurdin Ali, Syifaul Huzni, Ikramullah Ikramullah, Samsul Rizal

**Affiliations:** 1 Doctoral Program, School of Engineering, Universitas Syiah Kuala, Banda Aceh 23111, Indonesia; 2 Department of Mechanical Engineering, Faculty of Engineering, Universitas Samudra, Langsa 24416, Indonesia; 3 Department of Mechanical and Industrial Engineering, Universitas Syiah Kuala, Banda Aceh 23111, Indonesia

**Keywords:** zinc oxide (ZnO), oil palm empty fruit bunches (OPEFB), foamed polymer composites, automotive interior

## Abstract

The sustainable use of agricultural waste to generate valuable products while minimizing environmental burdens is increasing rapidly. Multiple sources of fibers have been intensively studied concerning their application in various fields and industries. However, few publications have extensively discussed the property’s performance of oil palm empty fruit bunches (OPEFB) composites. With main properties similar to composites currently listed for industrial applications, OPEFB is worth listing as a potential composite for industrial applications and non-structural material alternatives. OPEFB-reinforced polymer composites are expected to be applied to automotive interior parts. This study aims to determine the effect of adding zinc oxide (ZnO) and polyurethane on OPEFB-reinforced polymer composites for automotive interior parts. This composite was produced using the hand lay-up method with 70% resin, 15% OPEFB fiber, 15% polyurethane as a blowing agent, and four variations of ZnO at 5%, 10%, 15%, and 20%. The OPEFB particle sizes are 40, 60, 80, and 100, respectively. The composite was examined to determine mechanical, morphology, chemical, and thermal characteristics. It was observed that the addition of 20% ZnO caused ZnO agglomeration, weakening the interfacial bond between OPEFB particles, polyester, polyurethane, and ZnO filler. Overall, the results showed that adding ZnO and polyurethane to the composite increased tensile, compressive, flexural, and impact strength, as well as thermal stability with more significant values up to 160%, 225%, 100%, 100%, and 4.3%, respectively. This result depicted that the best composition was specimens with 15% ZnO and 149 microns OPEFB fibers particle size. It is considered a promising candidate to be applied in automotive interior components.

## 1. Introduction

Strict environmental regulations have been implemented in many countries to push many markets towards more environmentally friendly materials and processing. The production of synthetic fibers relies heavily on petrochemical-based resources, which are also rapidly depleted; they produce byproducts and hazardous waste, which are also a supporting force on the sustainability aspects of landfills. Although various industries and manufacturers use more environmentally friendly materials today, the automotive industry is the most influential [1]. It was thought that using natural fiber-reinforced composites in the automotive sector could offer an attractive alternative to these plastics due to technological, economic, and ecological reasons. Natural fiber-reinforced composites also have less risk during manufacturing processes, low emissions, and low abrasive properties, thereby reducing the possibility of damaging production equipment [2].

Furthermore, using natural fiber composites in the automotive industry will reduce vehicle weight and emissions. An estimated 25% reduction in vehicle weight would save 250 million barrels of unrefined petroleum [3]. Additionally, the advantages of natural fiber composites in the automotive industry are low density, sound insulation, acoustic properties, more accessible and faster manufacturing processes, environmental balance, improved accident performance with high stability, health considerations, and low cost [4]. The use of natural fibers in the automotive industry was primarily limited to non-structural parts, such as the interior trim of door panels, dashboards, rear racks, and seat upholstery [5]. 

Many natural fibers composites have been commonly used in interior components for commercial and passenger vehicles. Generally, natural fibers polymer composites are often used for the interior of cars. For example, dashboards produced using natural fibers composites on the BMW E-class [6]. The BMW 7 series uses door panels manufactured from BASF thermosetting acrylic copolymers and prepreg natural fiber mats. Audi also produces polyurethane door trim panels filled with a residual/hemp fiber mixture. Ford uses wheat straw as a filler with polypropylene to build injection molding storage and inner covers; in addition, Ford also uses soy-based polyurethane-based parts such as seat headrests, headliners, and chairs [3]. 

Currently, the sustainable use of agricultural waste to minimize environmental burdens while maintaining ecosystem sustainability is increasing rapidly. Many types of fiber’s natural sources have been intensively studied concerning their application in various fields and industries [7,8]. However, only a small number of publications have discussed extensively the physical, mechanical, and thermal performances of oil palm empty fruit bunches (OPEFB) [9,10,11]. As one of the most successful agro-industrial crops, and with overgrowing plantations around the world, improper disposal of OPEFB solid waste produced during the processing of palm oil fresh fruit bunches for palm oil production is a criticism directed at the palm oil industry [12]. Therefore, an innovative approach is required to turn OPEFB into a more valuable product that reduces the negative impact on the environment. OPEFB-reinforced polymer composites are expected to be an alternative to be applied to automotive interior panels. 

OPEFB-reinforced polymer composites have low impact strength, high-water absorption, and weight. Alternatively, a recent study finds that the finer OPEFB particles have increased composite tensile strength and thermal properties [13]. This finding placed OPEFB amongst the lists of potential composites since it shared almost similar tensile and modulus properties to coir, jute, sisal, hemp, and ramie, which have already been integrated into automotive applications [8,13]. OPEFB appears to have great potential to be applied for non-structural material alternatives. To furthermore elucidate this delinquent, adding filler to the composite is compulsory. Zinc oxide (ZnO) is one of the fillers reported to have improved the refractive index and thermal conductivity, binding properties, and anti-bacterial and ultraviolet protection of the material, which is beneficial for its more comprehensive application. It has also facilitated its mechanical and physical characteristics, as confirmed by previous studies that its addition to glass fiber composites leads to increased strength, flexibility, and thermal stability. It is also reported that it stabilizes ultraviolet light, affecting the weathering performance of wood-polyethylene composites by reducing their surface degradation [14,15,16]. 

Various nanoparticle fillers, including montmorillonite, silica, calcium carbonate, and aluminum oxide, have been reported to successfully improve the mechanical properties, thermal stability, electrical properties, gas barrier properties, and refractory properties of polymer matrix composite. Among various inorganic fillers, nano-ZnO has attracted much attention due to its unique physical properties as well as its low cost and wide application [17].

Polyurethane is a blowing agent that produces a type of plastic with a hollow cell-building structure. This material will accelerate the formation of foam, characterized by the appearance of tiny bubbles and colloidal stability, for the union of these bubbles of polyurethane to obtain lighter ingredients or products [18,19]. Polyurethane is also widely used in composite materials as a silencer. Tiuc et al. [20] examined the effect of adding textile waste to polyurethane foam on sound absorption ability. The addition of textile waste to polyurethane foam produces composite materials that can absorb sound up to twice as much as pure polyurethane foam. Adding ZnO fillers and polyurethane to OPEFB-reinforced composites is expected to improve mechanical, morphological, and thermal properties. Therefore, this study aims to determine the effect of adding ZnO and polyurethane on OPEFB-reinforced polymer composites for automotive interior parts applications.

## 2. Materials and Methods

### 2.1. Preparation of OPEFB Fiber and ZnO

The OPEFB was cleansed with water to remove adhering dirt and later soaked in water and 1% NaOH solution for approximately one day to remove the fat remaining on the surface. Then the OPEFB is dried under the sun to reduce the water content to ensure the OPEFB is dry enough to be processed into a fiber. The fibers are cut into 1–5 cm pieces using a CT 515 chopper and then mashed into fine fibers or particles. Subsequently, the fine fibers were filtered using a shave shaker machine to obtain 100, 80, 60, and 40 mesh sizes. Meanwhile, the ZnO used is synthetic, mainly applied commercially in the form of white flour or powder known as zinc white/calamine, and obtained from a chemical and composite distributor, Justus Sakti Raya Company, Jakarta, Indonesia.

### 2.2. Material Composition and Manufacture

**Figure 1:**
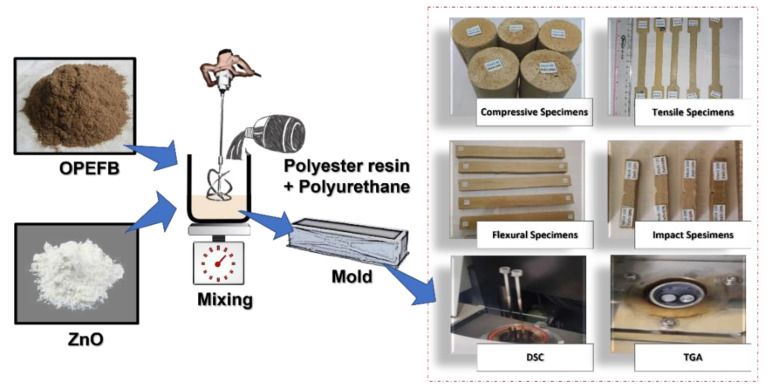
Material manufacturing process.

The composition of the material used in this study was based on Adlie et al. [21] findings that the best tensile strength for foamed polymer composites reinforced with OPEFB fiber was obtained with 70% polyester, 15% polyurethane, and 15% OPEFB. The foamed polymer composite material with OPEFB fiber and the addition of ZnO in this study was produced through the pouring/casting method, which involves pouring the mixture into a mold after thoroughly stirring in a mixing container. The polyester resin used as the matrix was later mixed with polyurethane as a blowing agent and different mesh sizes of OPEFB fiber at 40, 60, 80, and 100. It is important to note that the composition of each constituent material was based on the weight fraction, which includes 70% polyester, 15% polyurethane, and 15% OPEFB fiber. Meanwhile, the ZnO was added at 0%, 5%, 10%, 15%, and 20% of the combined material weight, while the catalyst used was the methyl ethyl ketone peroxide (MEKP). The specimens were made and fabricated using the hand lay-up method. The processes used in manufacturing the composite are presented in the following Figure 1.

### 2.3. Mechanical Test

The mechanical test was conducted using the RTF-1350 Series Tensilon Universal Testing Machine manufactured by A&D Company Ltd, Tokyo, Japan. It should be noted that the tensile test specimens were made and tested according to ASTM D-638. The compression test was based on the ASTM D-695 standard. Subsequently, the flexural test was in line with the ASTM D 790–92 standards for reinforced plastic materials and through the three-point bending method. These tests were conducted under ambient conditions, including a temperature of 25 °C and air humidity of 60% RH, and through a 5 kN load cell rating with a 5 mm/min test speed. Each test was conducted using five test specimens, after which the average value was used to determine the results. The impact strength was tested according to ASTM D5942-96 using the Charpy method at a temperature of 25 °C and air humidity of 60% RH to determine the energy needed to break the specimen. It is noteworthy that five samples were used for each composition, after which the average value of the results was determined.

### 2.4. Scanning Electron Microscopy (SEM)

This morphology of the foamed polymer composite with OPEFB fiber and ZnO was analyzed to determine the composition of the constituent materials, surface condition and texture, shape and size of the particles, as well as the bonds between the particles. A Scanning Electron Microscope (SEM) Hitachi SU 3500 model by Hitachi High-Technologies Corporation, Tokyo, Japan, with a voltage of 10 kV was applied to conduct this test. The sample is not conductive, suggesting it required a thin layer of gold coating before the observation.

### 2.5. Chemical Test

#### 2.5.1. Fourier Transform Infrared Spectroscopy (FTIR)

The chemical composition of the composites was investigated using FTIR analysis Shimadzu IR-Prestige 21 (Kyoto, Japan). The peaks were recorded at a wavelength of 400 to 4000 cm^−1^ to study the functional group and phase of the composites.

#### 2.5.2. X-ray Diffraction (XRD)

XRD patterns of all samples were examined on a Shimadzu 7000 (Kyoto, Japan), with X-ray wavelength CuKα (λ = 1.5406). Intensity at 2θ was recorded from 10 to 80°. 

### 2.6. Thermal Test

The thermal stability of the composite material was analyzed using thermogravimetry analysis (TGA) and differential scanning calorimetry (DSC) in line with the ASTM D3850 and ASTM 3418 standards. A 5 mg test sample was scanned at a heating rate of 100 C/min in a nitrogen atmosphere with a gas flow of 20 mL/min and a heating temperature ranging from 30 to 600 °C. The TGA and DSC tests were conducted using the Linseis Thermal Analysis Model Simultaneous Thermal Analysis (STA) PT1600 TG-DSC/DTA equipment manufactured by Linseis Messgeraete GmbH, Selb, Germany. The data for the TGA was presented in the form of a graph explaining the comparison of the heating temperature and the sample’s weight loss percentage. At the same time, the DSC chart result indicated the heat change to temperature rate.

## 3. Results and Discussion

### 3.1. Mechanical Properties Analysis

**Figure 2:**
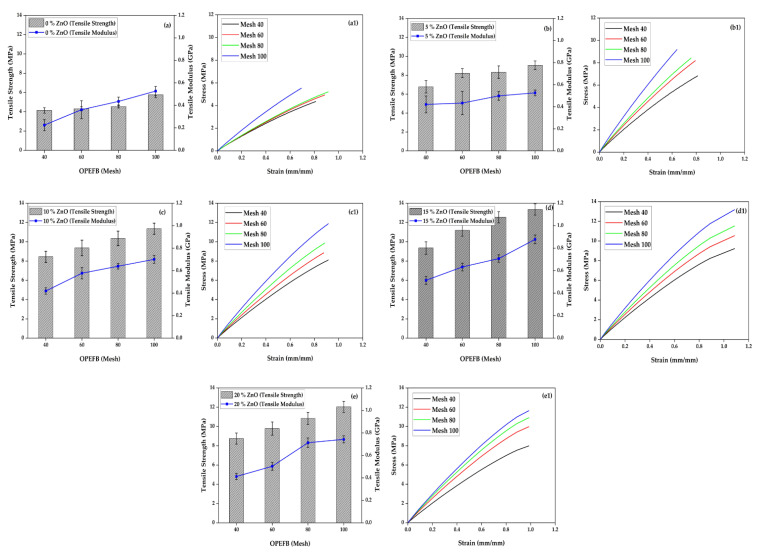
Tensile properties of foamed polymer composite at different OPEFB particle sizes and ZnO fillers, (**a**) 0% ZnO, (**b**) 5% ZnO, (**c**) 10% ZnO, (**d**) 15% ZnO, (**e**) 20% ZnO, (**a1**−**e1**) tensile stress–strain diagrams.

The comparison of tensile strength and modulus of foamed polymer composite material with OPEFB fibers and ZnO is shown in Figure 2, with variations in ZnO composition of 0%, 5%, 10%, 15%, and 20%, respectively, with OPEFB particle sizes, mesh 40, 60, 80 and 100. The graph shows that the addition of ZnO filler at 5%, 10%, and 15% percentage increase the tensile strength and modulus of the composite. However, the tensile strength decreased at 20% ZnO composition. Tensile strength increases with the smaller OPEFB particle size, where, with a mesh particle size of 100, the composite tensile strength becomes the highest among other OPEFB particle sizes. The addition of ZnO to the composite affects the tensile strength. An increase in tensile strength occurs in all variations of the composition of ZnO, ranging from 5–20% of the content (Figure 2). This result shows that adding ZnO to the composite can fill the cavity on the surface of the composite, both void and cavities that occur due to the effects of polyurethane reactions forming foam. 

The 15% ZnO composition showed the highest tensile strength with a value of 13.352 MPa, compared to the 5%, 10%, and 20% compositions. This composition of 15% ZnO (Figure 2d) uncovers the nearly perfect mixture between matrix (polyester), blowing agent (polyurethane), OPEFB fibers, and ZnO fillers. The bonding interface is appropriate, and the fibers are dispersed and evenly distributed throughout the surface of the composite. While in the composition of ZnO 20% (Figure 2e), clots occur, fibers and filling materials are not evenly distributed, the fibers experience pullouts from the matrix, and the bonds become saturated. All of these settings prompt the tensile strength of foamed polymeric composites to decrease. The results of this study are supported by Devaraju et al. [22], where an increase in the tensile strength of the test object took place due to the addition of 0.5% wt ZnO NP with an epoxy matrix combined with OPEFB random woven fibers. The specimen with 15% ZnO and 100 mesh size particles was found to have a better stress value than other compositions. The result is in line with the findings of [21,23,24] that a smaller size of fiber usually produces foamed polymer composite material with higher tensile strength. The ZnO addition was also observed to have contributed to the increment in the material’s tensile strength.

Figure 2(a1–e1) shows stress–strain diagrams of polymeric foam-reinforced composites added with ZnO that were tested at different sizes. The specimen with 15% ZnO and 100 mesh size particles had better stress and strain value than other compositions. Figure 2(d1) shows that the sample with 15% ZnO and 100 mesh particle size had the highest stress and strain values, with 13.352 MPa and 0.025 mm/mm, respectively.

The modulus tensile is one characteristic related to a material’s rigidity. Figure 2 shows the modulus value of the composite tensile increase along with the increase in tensile strength. The composite reaches its highest tensile modulus when adding 15% ZnO compared to 5%, 10%, and 20%. The particle size of OPEFB turned out to affect the value of the composite tensile modulus. The tensile modulus of the composite increases linearly as the size of the OPEFB particle decreases, as depicted in Figure 2a–e. The highest tensile modulus value is the composite with an addition of 15% ZnO and a mesh OPEFB size of 100, which is 0.878 GPa. The addition of ZnO in composites with up to 15% fiber content contributes to the high rigidity of the composite due to the excellent bond between ZnO, OPEFB particles, and polyester matrices. The tensile modulus value decreased at 20% ZnO content (Figure 2e) due to the saturation of OPEFB and ZnO particles; therefore, the bond between the composite materials became unstable. Anuar et al. [25] have investigated that the modulus tensile in the PP matrix composite with the addition of OPEFB fiber content up to 70% has increased the tensile modulus value. 

**Figure 3:**
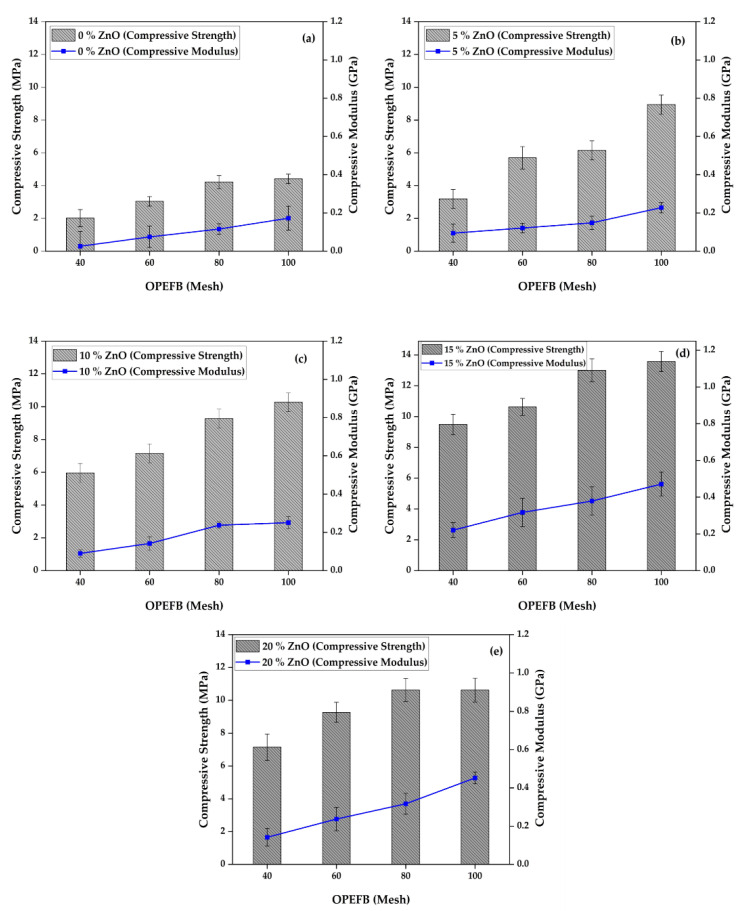
Compressive properties of foamed polymer composite at different OPEFB particle sizes and ZnO fillers, (**a**) 0% ZnO, (**b**) 5% ZnO, (**c**) 10% ZnO, (**d**) 15% ZnO, (**e**) 20% ZnO.

Figure 3 shows a comparison diagram of a foamed polymer composite’s compressive strength and modulus with variations of OPEFB fiber and ZnO filler. The test results also show the same phenomenon as the tensile strength test results, where adding ZnO to the composite with a composition of 5%, 10%, 15%, and 20% can increase its compressive strength. This result is similar to the findings of [26,27] that the hybridization of polyurethane foam bio-nano composites with OPEFB and nano-clay used as fillers enhanced the composite material’s compressive strength and thermal stability compared to the use of only OPEFB fiber in the bio-composites. Likewise, the OPEFB particle size, which shows that the mesh size of 100, the smallest particle size, can increase the compressive strength better than the foamed polymer composite without OPEFB fiber, and the particle size of the OPEFB mesh of 40, 60, and 80. It has been studied that increasing the volume fraction of date palm (DP) fibers reduces the compressive strength of mortar/DP NFC up to 5 MPa. The report’s results said that the compressive strength of the composite’s 5% DP fiber content was drastically lower than that of pure mortar [28]. 

It was discovered from Figure 3d that the best compressive strength was found in the specimen with 15% ZnO and 100 mesh size, as indicated by a stress value of 13.576 MPa. The compressive strength increased as more ZnO was added to the composite from 5–15% but reduced at 20%. This indication explains that it is possible to evenly disperse the fiber and ZnO in the matrix and maximize the surface area to ensure interactions between the filler and matrix phase. The ZnO was found to surrounded and filled the space between polyester resin, polyurethane, and fiber on the composite surface, indicating good bonding between the materials [29].

Compression modulus increases with the addition of ZnO to foamed polymer composites. Figure 3b–e shows that adding 5%, 10%, 15%, and 20% ZnO can increase the compression modulus of the composite. However, it slightly decreased the compression modulus value by adding a composition of 20% ZnO (Figure 3e). The highest value of compression modulus is found in composites with a composition of 15% ZnO with a particle size of OPEFB mesh 100, which is 0,471 GPa (Figure 3d). The larger OPEFB particle size also has a large cross-sectional area, requiring a more significant compressive load at the testing time. This particle size also causes the compression strength to increase while the strain decreases. This phenomenon is supported by studies that have been reported by Anuar et al. [30], stating that the compression modulus of OPEFB fiber is better than kenaf fiber. The size and length of OPEFB fiber are larger than kenaf fiber, capable of transferring the residual load.

**Figure 4:**
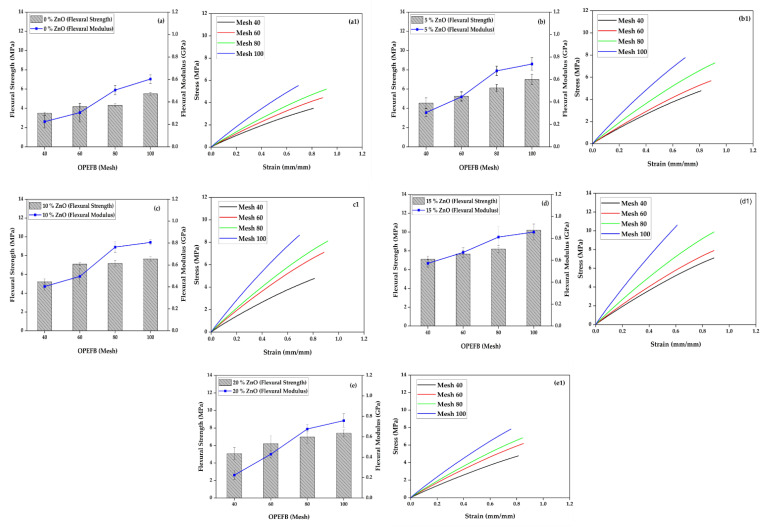
Flexural properties of foamed polymer composites at different OPEFB particle sizes and ZnO fillers, (**a**) 0% ZnO, (**b**) 5% ZnO, (**c**) 10% ZnO, (**d**) 15% ZnO, (**e**) 20% ZnO, (**a1**−**e1**) flexural stress–strain diagrams.

Figure 4 shows a comparison curve of the flexural strength of each ZnO and OPEFB fiber composition variation. The highest stress was recorded to be 10.195 MPa on 100 mesh OPEFB particle size at 15% ZnO compared to larger ones such as 40, 60, and 80. This result indicates that finer OPEFB particles can produce higher compatibility to increase the bond between individual particles. A study report found that pure OPEFB specimens had lower flexural strength than a sandwich hybrid structure consisting of a thin layer of OPEFB and two outer layers of hemp [31]. Sackey et al. [32] found that finer particle size fraction at the correct composition ratio had a significant impact on the particleboard’s internal bond strength, which is in line with the findings of this research. The addition of 15% ZnO filler was observed to have increased the flexural strength of the composite more compared to 5%, 10%, and 20%. Fine particles improve flexural properties better than larger particle sizes in all conditions. Fine particles can produce better compatibility to increase the bonding between individual particles [33,34]. These outcomes resemble previous studies investigating the mechanical properties of woven kenaf bast fiber/hybrid OPEFB reinforced with polyhydroxybutyrate bio-composite. They found the tensile and flexural strength of the reinforced bio-composite with 11 layers suitable as an alternative non-structural building material for several types of wood [34,35].

The flexural modulus of foamed polymer composites has increased along with the percentage of ZnO addition to composites. However, it slightly decreased at the composition of 20% ZnO (Figure 4a–e). The highest flexural modulus value is found in the composition of 15% ZnO, with a value of 0.857 GPa (Figure 4d). The composite flexural modulus increases linearly as the flexural strength increases. This result shows that the stress and strain in the bending test have increased equally and are directly proportional. The increase in the flexural modulus value of the composite is also in accordance with the decrease in OPEFB particle size, where at all particle sizes of mesh 40, 60, 80, and 100, and all ZnO compositions, there is an increase in the flexural modulus value of the composite. The highest value of flexural modulus is found in the particle size of OPEFB mesh 100. Figure 4 also displays the flexural strength comparison curves of ZnO and OPEFB fibers at each variation. The figure also compares the stress–strain values of every OPEFB particle size of 40, 60, 80, and 100 mesh. The highest stress value of 10.195 MPa, and strain of 0.012 mm/mm, was found at mesh 100. This result indicates that finer OPEFB particles can produce higher compatibility to increase the bond between individual particles. The flexural strain value of the foamed polymeric composite is also directly proportional to the flexural stress, where adding ZnO to the composition of 5%, 10%, 15%, and 20% increases the strain value. OPEFB particle size also affects the strain; the smaller the OPEFB fiber size, the greater the strain that arises (Figure 4(d1)).

Bilisik et al. have reported that topography and chemical elements on the surface of the fibers have an essential effect on the interface bond between the matrix and the fibers that make up the composite [36]. There was an interesting relationship in the results of this bending test where flexural strength and flexural modulus experienced an increase in values that were directly proportional to the increase in the number of ZnO compositions and the reduction of OPEFB particle size. This trend can be related to the rigid basic properties of ZnO and OPEFB that affect the rigidity of the composite.

**Figure 5:**
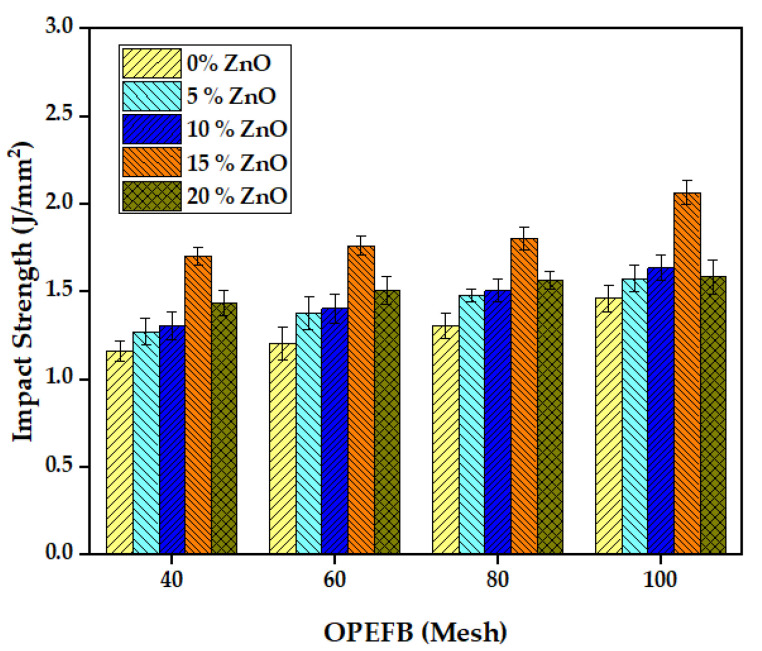
Impact strength comparison of foamed polymer composites at different OPEFB particle sizes.

The impact test results in Figure 5 showed that adding 15% ZnO increased the impact strength of the composite material compared to the specimen without ZnO. A similar tendency was observed at 5% and 10%, while a reduction was recorded at 20%. Moreover, the OPEFB particle size also affected the impact strength [37], with the smaller size at 100 mesh observed to have a higher increase than the bigger sizes, such as 40, 60, and 80. Kakou et al. [38] have reported a significant increase in impact strength of 40% over 30% in composites with OPEFB fiber. More OPEFB fiber content plays an essential role in the toughness and impact strength of high-density polyethylene (HDPE) composite materials. The highest impact strength value was 2.067 J/mm^2^ at 100 OPEFB particle size and 15% ZnO, while the lowest was 1.164 J/mm^2^ at 40 OPEFB particle size and 0% ZnO. The findings of [25,31] supported that adding ZnO to glass fiber composites increased the impact strength and flexibility and ensured good thermal stability. The comparison of the current findings with previous reports of the mechanical properties of OPEFB and its composites is shown in Table 1.

### 3.2. Morphological Studies

It is known that the dispersion of fillers in the polymer matrix composite has a significant effect on the mechanical properties of the composite. Spreading inorganic fillers in thermoplastic matrices is a challenging process, simply because micro/nanoparticles fillers mixture in polymer composites strongly tend to agglomerate. As a result, the homogeneous dispersion of micro/nanoparticles on a thermoplastic/thermosetting matrix becomes complicated. A study published that the nanoparticles (5 wt% nZnO) were well dispersed in the matrix. In contrast, the microparticles (5 wt% mZnO) exhibited a broad size distribution and greatly affected the mechanical properties of the composite. The results of this research study indicate that incorporating ZnO particles into the iPP matrix can significantly improve the composite’s mechanical properties. The mechanical characterization results showed that the tensile strength and tensile modulus of iPP/nZnO composites were higher than those of iPP/nZnO composites without fillers and even higher than those of iPP/mZnO composites. [42].

**Figure 6:**
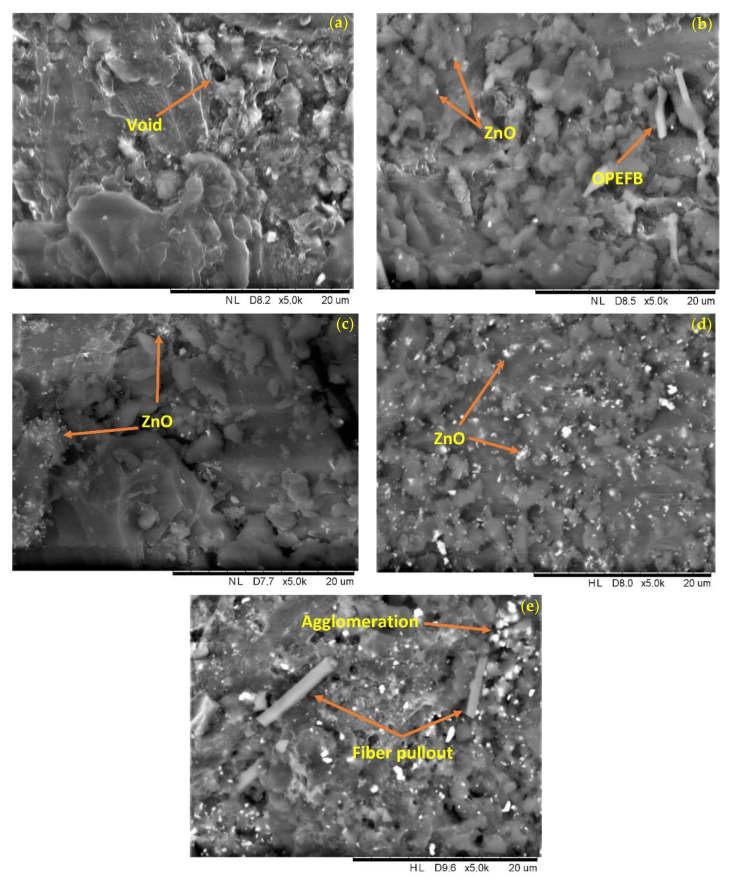
Surface morphology of foamed polymer composites at different ZnO fillers, (**a**) 0% ZnO, (**b**) 5% ZnO, (**c**) 10% ZnO, (**d**) 15% ZnO, (**e**) 20% ZnO.

The SEM image in Figure 6a shows the surface of the OPEFB fiber-reinforced foamed polymer composite without ZnO (0%) filler, produced based on the best composition stated in [21], which includes 70% resin, 15% OPEFB fiber, and 15% polyurethane. The SEM image shows that many voids are still forming in the composite. Adding ZnO as a filler is expected to fill the voids and cavities in the composite to improve the mechanical, physical, and thermal properties supported by SEM images. As previous research reports stated, adding nano clay to the polyurethane bio-composite reinforced with the fiber was well-distributed and homogeneously mixed with the matrix [27]. The cavities formed in the composite—a result of the reaction of the polyurethane (blowing agent) and the bond between the polyester (which serves as the matrix) and OPEFB fiber—were found to be suitable. This circumstance allowed the fiber to spread evenly and homogeneously fill the cavities of the composite. 

Figure 6b,c indicate the surface of the specimens produced using 5% and 10% ZnO as filler, respectively, and it was discovered that the ZnO did not spread evenly throughout the surface. Meanwhile, the 15% ZnO specimens in Figure 6d showed an even distribution and an interfacial bond between the matrix, well-established fiber, and the filler. This state signifies that the mixture of the composite materials is uniform or homogeneous, and this is in line with the previous results [43,44,45], which showed an increment in the mechanical properties such as tensile, compressive, flexural, and impact strengths compared to 5% and 10% (Figure 2, Figure 3, Figure 4 and Figure 5). 

The findings from the 20% ZnO addition are presented in Figure 6e. It was discovered that the ZnO is not dispersed throughout the composite surface, and the material is agglomerated, forming many voids. This disarray implies that the bond between the particles that make up the composite, including the matrix, OPEFB fiber, and ZnO, has become weak. The SEM image also shows that the agglomeration and fiber pullout occurred due to this weak interfacial bond, thereby causing a reduction in the mechanical characteristics of the foamed polymer composite [46]. The phenomenon was confirmed by the findings of [25,27], where breakage and pullout occurred when OPEFB composition was set above 70%. Subsequently, the mixture of the particles used for the composite had become saturated, the interfacial bond was weakened, and agglomeration occurred, thereby deforming the fiber distributed evenly on the matrix surface.

### 3.3. Chemical Analysis

#### 3.3.1. FTIR

**Figure 7:**
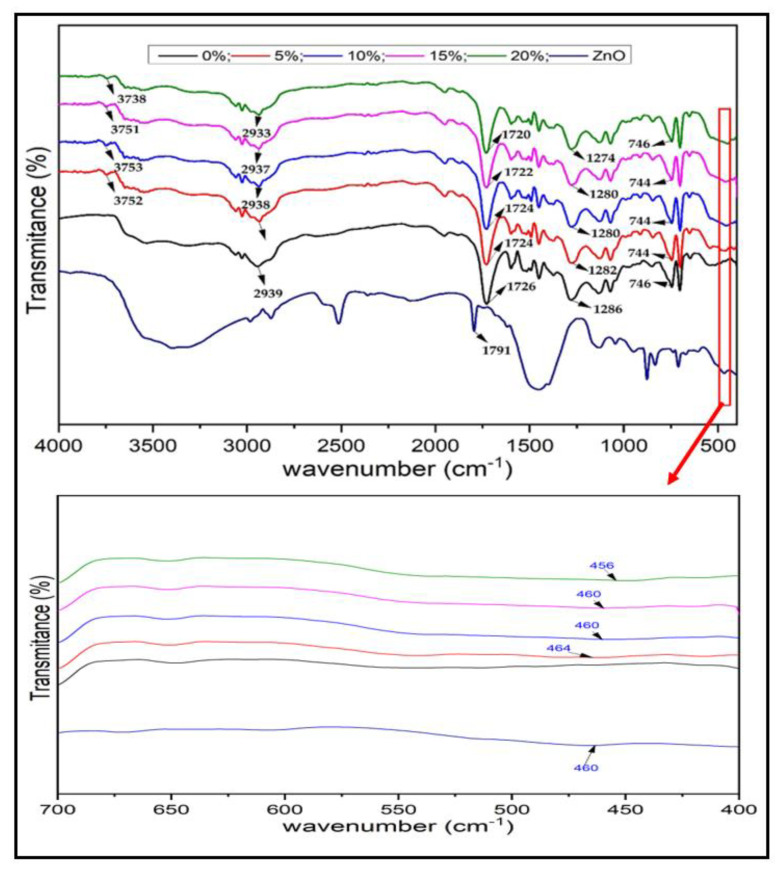
FTIR spectra of foamed polymer composites at different ZnO fillers.

The FTIR spectrum of foamed polymer composites at different ZnO fillers is shown in Figure 7. The diffractogram of Fourier transform infrared (FTIR) characterization results shows that five dips are identified, as presented in Table 2, which explains that a functional group (stretching OH) at wave number 3745 cm^−1^ was identified in every upsurge of the ZnO element (5%, 10%, 15%, and 20%) in the composite. On the contrary, the dip was not found at 0% of the ZnO, indicating the absent ZnO element in the composite. Intensification of the ZnO element does not significantly influence its dip due to the absorption of the infrared wave in the composite. However, the occurrence of deep shifts in each increase of the ZnO element. Deep shifts can be caused by several factors, including the transition of electrons in ZnO mixed with composites due to the influence of photons fired on the sample during the characterization process. The pure ZnO spectrum at the very bottom of the graph is a reference to justify the peak absorbent presence of ZnO in the composite. Based on observations, ZnO absorption peaks are found in wave numbers 456 to 464 cm^−1^ [46]. However, if observed at a glance, looking at the wave range or as a whole, it does not describe the significant characteristics of ZnO, so it is assumed to be influenced by several factors including the ZnO used is not pure ZnO but technical ZnO, so that the ZnO phase contained in the composite is still discrete and does not undergo deformation or phase change, the temperature during the synthesis process of mixing ZnO with composites is still lower than the synthesis temperature to form technical ZnO, because ZnO does not undergo phase changes during the mixing process with composites so that the parts characterized by FTIR are only partial or even absent. Based on this assumption, the FTIR graph from ZnO does not describe the existence of ZnO significantly but more dominantly describes the vibration of streching and bending that occurs in composite FTIR results in general.

#### 3.3.2. XRD

**Figure 8:**
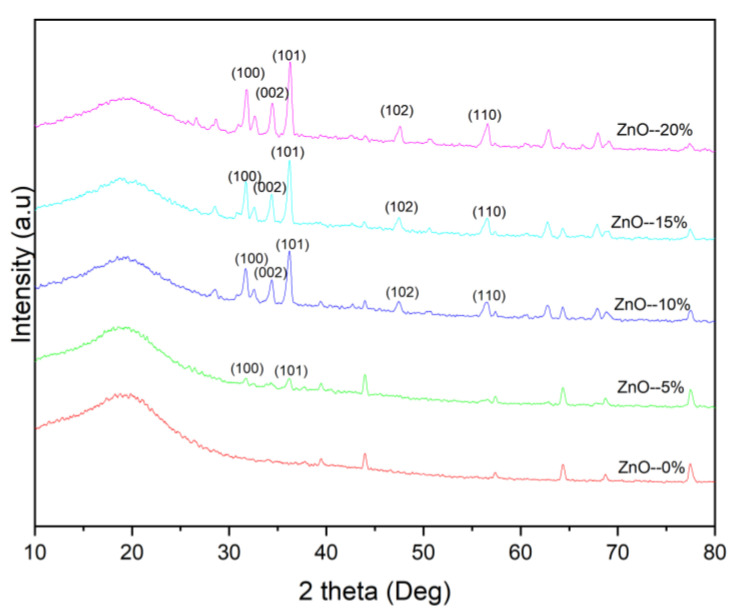
X-ray diffraction patterns of foamed polymer composites at different ZnO fillers.

**Figure 9:**
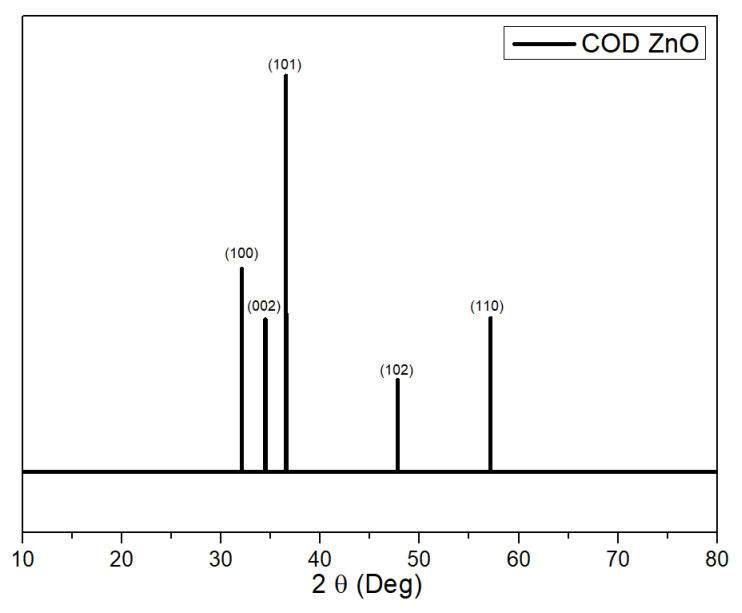
Crystallography of Database (COD) code 1011258.

From the results of the X-ray diffraction (XRD) diffractogram displayed in Figure 8, the sample is polycrystalline and confirmed the presence of ZnO elements characterized by the presence of ZnO diffraction peaks following the Crystallography of Database (COD) code 1011258 in Figure 9.

The diffraction peak is increasingly visible as the ZnO element increases in the composite. This peak can be observed by increasing the intensity value in each diffraction field as the ZnO element increases. The identified area of diffraction is (100), (002), (101), (102), (110); in addition to the apex peaks of ZnO, other diffraction peaks are assumed to be noise formed from the amorphous phase. From the diffraction fields, it can be determined which crystallite size is presented in Table 3. 

From Table 3, the crystallite size tends to decrease along with the increasing percentage of the element ZnO, and the lattice parameters tend to increase as the percentage of ZnO elements increases. This condition is assumed to be caused by crystalline defects that appear during the synthesis process of composite manufacturing. There was an indication of ZnO elements amalgamation with other elements, thereby changing ionic bonds between elements. Subsequently, this change causes deviations in the position of the element O to Zn, known as lattice distortion. Moreover, due to lattice distortion, it is assumed that lattice expansion in the crystallite—which expands the distance between lattices—does not affect the crystallite size. Nevertheless, it affects the crystal’s distance between atoms (hopping length) or sub-disability. The result of the expansion of the lattice causes imperfections of a crystal, known as the value of the crystal defect (strain), as portrayed in Table 3. 

### 3.4. Thermal Analysis

**Figure 10:**
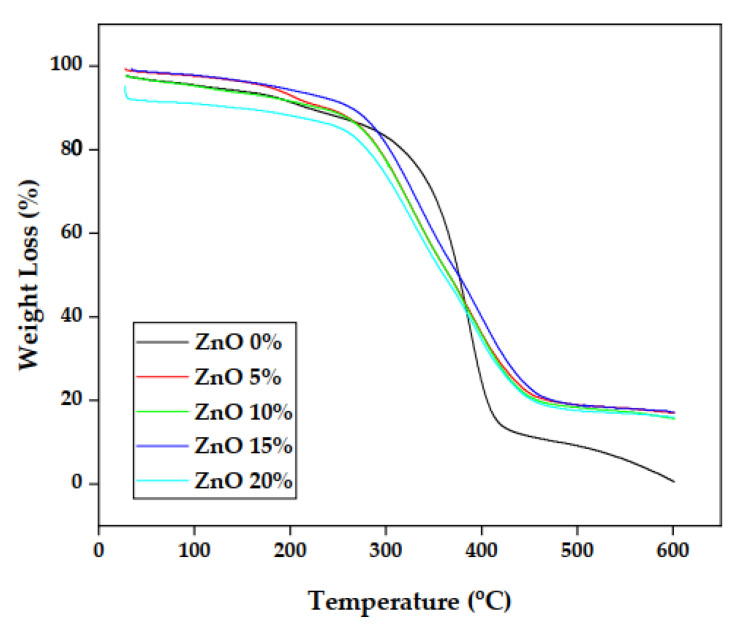
Graph of thermogravimetry analysis (TGA) at each variation of ZnO.

Figure 10 shows the most widely applied thermogravimetric curve (TGA) to analyze the thermal properties of a material with a focus on foamed polymer composites with OPEFB fiber filled with ZnO at 0%, 5%, 10%, 15%, and 20%. This TGA curve provides information on the rate of mass change in temperature. The heating process commences at 30 °C, where the sample loses a small amount of its mass due to water evaporation. At the same time, degradation was characterized by a decrease in mass that occurs at temperatures above 300 °C (T_onset_). The sample with 0% ZnO achieved the highest T_onset_ at 285.88 °C, while maximum degradation occurred at 436.32 °C (T_endset_) in 15% ZnO with a weight loss of up to 76.42%.

The minimum degradation temperature (T_endset_) of 402.61 °C and the highest mass reduction of 96.96% was recorded for the composite without ZnO filler. The composite further decomposed to produce a residue in the form of carbon at a temperature range of 460–600 °C. This condition indicates that the addition of the ZnO increased the thermal stability of the composite [17,47,48]. The complete TGA results are presented in the following Table 4.

**Figure 11:**
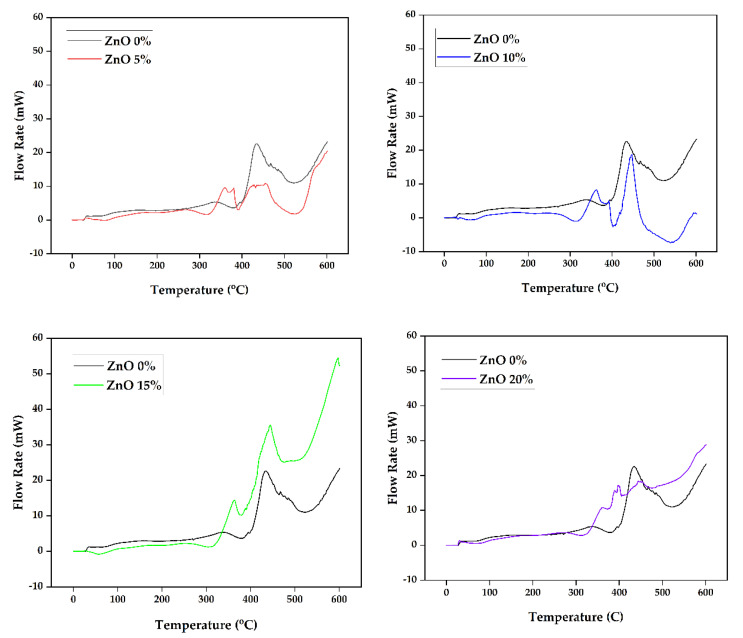
Graph of differential scanning calorimetry (DSC) at different variations of ZnO.

The results of the thermal test conducted using differential scanning calorimetry (DSC) are presented in Figure 11. The test was initiated at a glass temperature of 20 °C, and the curve continued to slope as the temperature rate increased to 20 °C/min. The process occurs with the five variations of the ZnO observed to have a curve pattern with a similar tendency. A crystallization reaction occurs in the composite at a temperature range of 342–393 °C. The reaction at this temperature is known as an exothermic reaction (T_onset_). The lowest T_onset_, 342.80 °C, was found at 0% ZnO, while the highest, 393.55 °C, was at 15% ZnO. The exothermic reaction peaked temperature (T_peak_) was 402.17 °C in the composite with 15% ZnO and the lowest T_peak_, 373.34 °C, was also in the composite without ZnO (0%). At this crystallization temperature, the material releases a certain amount of heat by changing the phase of the material into crystals. The complete results of the DSC test are listed in Table 5.

The process after the crystallization or exothermic reaction occurs in the composite material is the melting process, which occurs at a melting temperature (T_endset_). During this phase, the material requires heat to melt the entire material, known as an endothermic reaction. The lowest melting temperature was found in composite materials without ZnO filler at 384.40 °C, while the highest was at 424.35 °C in 15% ZnO. The composite material begins to degrade at temperatures above 425 °C and decomposes at 600 °C. These are similar to the DSC test results from previous studies where the non-isothermal crystallization behavior of iPP/mZnO and iPP/nZnO composites was investigated using DSC. The study showed that the interfacial interaction between iPP and ZnO increased the crystallization temperature at 5% wt% ZnO content [27,49]. The highest melting temperature of 15% ZnO was found for 100-mesh particle size, associated with the cellulose change where the amorphous was degraded to a crystalline structure at this reaction. Increasing the ZnO fraction in composite seems to increase the crystallization and melting temperature [48,50], as there are up to 40 °C temperature differences between T_endset_ of 0% and 15% ZnO.

The DSC test results indicate that adding ZnO fillers to this foamed polymeric composite can increase the thermal stability of the composite. ZnO in polymeric foamed composites also has a significant heterogeneous nucleation effect on composite materials. SEM imagery results supported the argument, revealing an unfluctuating dispersion of ZnO on the surface of the composite material. A composition of 15% ZnO is better than the other.

## 4. Conclusions

The analysis of the mechanical properties of foamed polymer composites with different OPEFB particle sizes of 40, 60, 80, and 100 and added with different variations of ZnO at 0%, 5%, 10%, 15%, and 20% showed that the best tensile, compressive, impact and flexural strengths were obtained at the specimen with 15% ZnO and 100 mesh size of OPEFB. Adding up to 15% ZnO to the foamed polymer composite successfully increased the mechanical properties; however, a reduction was observed at a higher percentage. Moreover, the SEM analysis showed that the addition of 20% ZnO caused ZnO agglomeration, which led to a weak interfacial bond between OPEFB particles, polyester, polyurethane, and ZnO filler. It is because the OPEFB particles and ZnO are not well dispersed on the entire surface, leading to a heterogeneous mixture of each material. 

The FTIR characterization shows t a functional group (stretching OH) at wave number 3745 cm^−1^ was identified in every upsurge of the ZnO element (5%, 10%, 15%, and 20%) in the composite. On the contrary, the dips was not found at 0% of the ZnO, indicating the absence of a ZnO element in the composite. The XRD diffraction peak is increasingly visible as the ZnO element increases in the composite, and this can be observed by increasing the intensity value in each diffraction field as the ZnO element increases. The crystallite size tends to decrease along with the increasing percentage of the element ZnO, and the lattice parameters tend to increase as the percentage of ZnO elements increases.

ZnO is one of the hydrophobic materials; consequently, it is widely applied to water-repellent components. Utilizing ZnO as fillers in the composite would diminish the high-water absorption properties of OPEFB fibers. Due to its good binder properties, ZnO can bind composite constituent materials well, such as matrices (polyester resins), polyurethanes, and OPEFB fibers. Adding ZnO with the exact composition can also improve tensile, compressive, and bending properties. This argument is supported by the results of SEM images, which show that at a composition of 15% ZnO and a particle size of OPEFB mesh 100 offers an even spread on the composite surface, the interface bonds between matrices, fibers, and fillers are well intertwined, ZnO is evenly distributed in the composite; this indicates that the mixture of composite constituent materials is homogeneous. The impact properties have also increased because ZnO’s primary properties are hard and brittle; therefore, adding ZnO to the OPEFB fiber composite can increase the impact strength. With a melting point reaching 1975 °C, adding ZnO as fillers can also improve the composite’s thermal stability. 

The thermal stability test was conducted using TGA, and the result revealed that the highest initial mass degradation of the composite was recorded for 0% ZnO at 285.88 °C (T_onset_). The highest mass degradation temperature (T_endset_) was found at 15% ZnO at 436.32 °C with a weight loss of up to 76.42%. While the lowest, 402.61 °C, with a weight loss of up to 96.96%, was at 0% ZnO. The composite was observed to have decomposed up to a temperature of 600 °C. Moreover, the exothermic reaction was evaluated using DSC, and the lowest was discovered to have occurred at 342.80 °C (T_onset_) in the minor component of 0% ZnO, while the highest, 393.55 °C, was at 15% ZnO. The highest exothermic reaction peak temperature (T_peak_) was also found at 15% ZnO, while the lowest was at 0% ZnO.

Furthermore, the lowest crystallization temperature (T_endset_), 384.40 °C, was recorded in the composite without ZnO filler, while the highest, 424.35 °C, was with a 15% ZnO filler composition. Using ZnO as a filler in OPEFB foamed polymer composites can improve thermal stability. The composite with 100 mesh OPEFB and 15% ZnO was generally found to be the best composition to obtain optimal composite performance and was considered a promising candidate for automotive interior components. 

## Figures and Tables

**Table 1:** Comparison of the current findings with previous reports.

Fiber		Matrix	Tensile Strength (MPa)	Tensile Modulus (GPa)	Flexural Strength (MPa)	Flexural Modulus (GPa)	Impact Strength	Ref.
OPEFB (10–20 mm length)	0 Vf	Epoxy	60	1.38	98	3.31	-	[39]
	5 Vf		30	1.43	41	3.29	-	[39]
	10 Vf		26	1.39	52	3.27	-	[39]
	15 Vf		25	1.37	42	3.3	-	[39]
	20 Vf		26	1.33	48	3.09	-	[39]
OPEFB	-	Epoxy	24	0.9	-	-	19 KJ/m^2^	[40]
OPEFB (40 wt%.)	-	Phenol-formaldehyde	10	0.5	10	2.1	25 KJ/m^2^	[41]
OPEFB	100 mesh	Polyester + polyurethane + 15% ZnO	13	0.87	10	0.85	2 J/mm^2^	This study

**Table 2:** Functional group of ZnO.

ZnO (%)	Wave Number (cm^−1^)	Functional Group
0	737	Stretching (C-N)
	1284	Bending (C-O)
	1716	Bending (CH_3_)
	2936	Stretching (OH)
5	745	Stretching (C-N)
	1275	Bending (C-O)
	1728	Bending (CH_3_)
	2931	Stretching (OH)
	3747	Stretching (OH)
10	747	Stretching (C-N)
	1280	Bending (C-O)
	1728	Bending (CH_3_)
	2938	Stretching (OH)
	3745	Stretching (OH)
15	759	Stretching (C-N)
	1275	Bending (C-O)
	1728	Bending (CH_3_)
	2938	Stretching (OH)
	3742	Stretching (OH)
20	741	Stretching (C-N)
	1272	Bending (C-O)
	1728	Bending (CH_3_)
	2938	Stretching (OH)
	3745	Stretching (OH)

**Table 3:** Crystallite size and lattice parameters.

ZnO (%)	HKL	Xc	FWHM	λ (Å)	a (Å)	±∆a	t (nm)	±∆t	Average t (nm)	Strain
0	0	0	0	0	0	0	0	0	0	0
5	100	31.69	0.33	1.5406	2.82	0.023	24.07	0.525854	22.74	0.020
	101	36.14	0.45	1.5406	3.51	0.018	17.65	0.273811		0.024
	110	57.4	0.3	1.5406	2.27	0.002	26.48	0.127171		0.010
10	100	31.68	0.41	1.5406	2.82	0.017	19.38	0.317344	16.52	0.025
	002	34.34	0.42	1.5406	5.22	0.026	18.92	0.279207		0.024
	101	36.16	0.46	1.5406	3.51	0.011	17.27	0.160802		0.025
	102	47.44	0.55	1.5406	4.28	0.017	14.44	0.232438		0.022
	110	56.45	0.63	1.5406	2.30	0.012	12.61	0.298221		0.020
15	100	31.7	0.34	1.5406	2.82	0.008	23.37	0.191455	17.81	0.021
	002	34.3	0.41	1.5406	5.22	0.021	19.38	0.228555		0.023
	101	36.18	0.41	1.5406	3.51	0.011	19.38	0.180506		0.022
	102	47.43	0.58	1.5406	4.28	0.017	13.70	0.220374		0.023
	110	46.47	0.6	1.5406	2.76	0.019	13.24	0.365679		0.024
20	100	31.79	0.35	1.5406	2.81	0.014	22.70	0.310832	17.18	0.021
	002	34.42	0.44	1.5406	5.21	0.016	18.06	0.160272		0.025
	101	36.25	0.45	1.5406	3.50	0.007	17.65	0.109847		0.024
	102	47.52	0.55	1.5406	4.28	0.030	14.44	0.407413		0.022
	110	56.51	0.61	1.5406	2.30	0.014	13.02	0.369957		0.020

**Table 4:** Results of the thermogravimetric analysis (TGA) test.

Material	T_onset_ (°C)	Midpoint (°C)	T_endset_ (°C)	Weight Loss (%)
Composite + 0% ZnO	285.88	365.85	402.61	96.96
Composite + 5% ZnO	236.67	340.21	425.92	82.06
Composite + 10% ZnO	259.35	342.25	430.93	81.92
Composite + 15% ZnO	250.20	345.45	436.32	76.42
Composite + 20% ZnO	245.56	342.26	427.29	82.64

**Table 5:** Differential scanning calorimetry (DSC) test results.

Material	T_onset_ (°C)	T_peak_ (°C)	T_endset_ (°C)
Composite + 0% ZnO	342.80	373.34	384.40
Composite + 5% ZnO	364.88	379.51	415.69
Composite + 10% ZnO	380.18	391.84	417.51
Composite + 15% ZnO	393.55	402.17	424.35
Composite + 20% ZnO	361.51	376.50	399.73

## Data Availability

Currently, data is not shared.
